# Estimating braking and propulsion forces during overground running in and out of the lab

**DOI:** 10.1371/journal.pone.0330042

**Published:** 2025-09-04

**Authors:** Lauren M. Baker, Fabian C. Weigend, Krithika Swaminathan, Daekyum Kim, Andrew Chin, Daniel E. Lieberman, Conor J. Walsh

**Affiliations:** 1 John A. Paulson School of Engineering and Applied Sciences, Harvard University, Boston, Massachusetts, United States of America; 2 School of Mechanical Engineering, Korea University, Seoul, Republic of Korea; 3 Department of Human Evolutionary Biology, Harvard University, Cambridge, Massachusetts, United States of America; Aston University, UNITED KINGDOM OF GREAT BRITAIN AND NORTHERN IRELAND

## Abstract

Accurately estimating kinetic metrics, such as braking and propulsion forces, in real-world running environments enhances our understanding of performance, fatigue, and injury. Wearable inertial measurement units (IMUs) offer a potential solution to estimate kinetic metrics outside the lab when combined with machine learning. However, current IMU-based kinetic estimation models are trained and evaluated within a single environment, often on lab treadmills. The transferability of these treadmill-trained models during overground running in and out of the lab is underexplored, and the individualization and validation of such models remain a challenge. Toward bridging this gap, we trained a generalized model on treadmill data of 15 recreational runners and evaluated braking and propulsion force estimates during overground running in and out of the lab. We explored fine-tuning with individual data from lab-based overground running to quantify model performance improvements with individualization. The generalized and fine-tuned models were extrapolated to outdoor running for a subset of five participants, and estimates were compared to lab-based overground measurements. Evaluating the generalized model with a leave-one-out cross validation yielded overground braking and propulsion force root mean squared error of 4.3 ± 1.1 % bodyweight (%BW). Fine-tuning this model with eight strides reduced error to 2.6 ± 0.5 %BW. Outdoor force predictions from the fine-tuned model better aligned with expected linear trends between braking/propulsion impulses and speed than the generalized model. These results provide insights into the accuracy and applicability of IMU data-driven models for braking and propulsion estimation during overground running, facilitating the development of practical, individualized biomechanical analysis tools for real-world use.

## Introduction

Running is a widely practiced form of exercise, with most recreational and competitive running taking place outdoors on roads, trails, and tracks [1]. Yet, much of the research on running biomechanics is conducted on treadmills in controlled laboratory settings [2]. To better understand biomechanical factors related to performance, fatigue, and injury development, we need accurate estimates of biomechanical metrics in real-world running environments outside the lab.

Wearable sensors are increasingly being used to study movement patterns in such settings [3–8]. Among biomechanical metrics of interest, ground reaction forces (GRFs) have garnered significant attention due to their hypothesized relationships with running-related injuries [9–11] and performance [12,13]. While instrumented pressure insoles can estimate GRFs [14–16], their impact on the foot-shoe interface and inability to capture distinct directional GRF components limit their utility for comprehensive kinetic analysis. Alternatively, inertial measurement units (IMUs) offer a less intrusive and more accessible option for runners. Research has shown that acceleration, angular velocity, and orientation measurements from IMUs combined with machine learning models enable estimates of kinetic metrics, such as GRFs, during running [17].

Training these models often requires large datasets, with individual-specific data typically yielding higher accuracy than generalized models [18]. However, collecting sufficient data for individual models can be challenging [19,20]. Fine-tuning, a technique that adapts generalized models with individual-specific data [21], could address data collection challenges, but the individual data requirements for GRF estimation from IMUs during running remain unclear. Furthermore, understanding the trade-offs between model accuracy and sensor configurations is essential for practical applications, as minimizing the number of sensors is key to enabling real-world use outside the lab [22].

In addition to understanding data requirements, the validation of these models presents another challenge. Kinetic estimation models in prior literature are trained and evaluated within a single environment, often on lab treadmills. The transferability of these models to overground running, whether in controlled lab settings or outdoor conditions, is underexplored and may be challenging due to biomechanical discrepancies between treadmill and overground running. Lab-based comparative studies of treadmill and overground running have reported differences in sagittal plane kinematics at foot contact as well as propulsion force, the positive and posterior component of the anterior-posterior GRF (AP-GRF) [23]. These kinematic and kinetic differences, along with known relationships between sagittal segment angles and braking forces, the negative and anterior component of the AP-GRF [24,25], highlight the need to understand how IMU data from treadmill running maps to braking and propulsion estimates during overground running. Although prior research has estimated braking and propulsion forces from IMU data-driven models in lab environments [18,20,26–29], these metrics have not yet been estimated during overground running outside the lab. Real-world estimation of AP-GRF metrics may help assess both running-related injury risk and performance capacity, given the association between high braking forces and injury [10], and the role of propulsion in endurance performance [13].

To improve the translation of IMU-based AP-GRF estimation models from laboratory to real-world running environments, this paper aims to narrow two gaps in the field: (1) model evaluation across running environments and (2) model requirements for fine-tuning and minimal sensorization. Specifically, this work:

- Develops a generalized model for estimating braking and propulsion forces during overground running from IMU data collected during treadmill running,
- Characterizes fine-tuning data requirements by examining the tradeoff between the number of strides collected and model accuracy,
- Validates the applicability of a treadmill-trained model to predict braking and propulsion forces in different environments, from lab to outdoors, for a subset of participants,
- Investigates optimal sensor configurations to minimize the number of IMUs while maintaining model accuracy, enabling broader adoption in outdoor settings.

## Materials and methods

### Study participants and protocol

**Fig 1:**
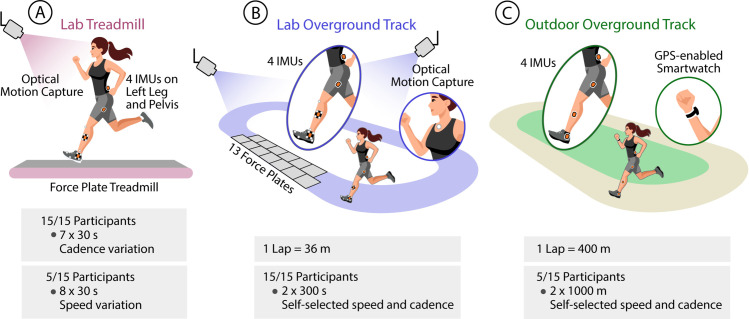
Data collection. IMU data were collected in three settings: **(A)** lab treadmill, **(B)** lab overground track, and **(C)** outdoor overground track. In the lab, the anterior-posterior component of measured GRFs provided braking and propulsion forces. We also collected optical motion capture data in the lab and GPS from a smartwatch outside the lab for synchronizing data, identifying straightaways, and calculating speeds.

Fifteen healthy adult volunteers (7 females; age: 27.7 ± 3.9 yr, height: 1.74 ± 0.09 m, mass: 72.8 ± 14.2 kg, mean ± standard deviation) participated in this study. This study builds on a previous dataset [25] comprising IMU and GRF data from 10 participants running on an instrumented treadmill and a 36-m overground oval track embedded with force plates along one straightaway, measuring 12.25 m. We expanded the dataset by collecting data from five additional participants following the same lab protocol, including additional treadmill speeds and overground running on a 400-m outdoor oval track (Gordon Track, Harvard University, Boston, MA) (Fig 1). The five new participants were recruited from 28 May 2024 to 19 June 2024. Inclusion criteria included running for recreational exercise, an ability to continuously run for 45 minutes, and no musculoskeletal injuries or disorders. The Harvard Longwood Medical Area Institutional Review Board (IRB) approved the study under protocol #22086. All research was performed in accordance with IRB-approved guidelines and regulations, and all participants provided written informed consent.

As part of the lab treadmill protocol, all participants completed seven 30-s trials at 3 m/s. Two of these seven trials were completed at a self-selected cadence, and five trials used a metronome to mandate cadences at 150, 160, 170, 180, or 190 steps per minute. A subset of five participants completed an additional eight 30-s trials at 2, 2.5, 3.5, or 4 m/s at either a self-selected cadence or a metronome-mandated cadence of 170 steps per minute. Short breaks were provided in between trials. On average, 405 ± 22 treadmill strides were collected from the cadence-variation trials for each participant, with an additional 427 ± 29 treadmill strides from the speed-variation trials for each of the five subset participants.

On the lab overground track, all participants completed two 5-min continuous running trials with a short break in between trials. Participants were instructed to run at a comfortable self-selected speed and cadence for both trials. During the second trial, participants were also instructed to either increase or decrease their speed every minute. On average, 78 ± 8 overground strides on force plates were collected for each participant. Including the non-instrumented side of the track, participants ran across 85 ± 11 straightaways (175 ± 22 strides).

On the outdoor overground track, a subset of five participants completed two 1-km (2.5 laps) continuous running trials with a short break in between trials for a total of 10 passes along the track’s 100-m straightaways (369 ± 36 strides). Participants were instructed to run at a comfortable self-selected speed and cadence for both trials, similar to the lab overground collection. During the second outdoor trial, participants were also instructed to either increase or decrease their speed every 200 m, as denoted by track markings.

### Instrumentation and data pre-processing

**Fig 2:**
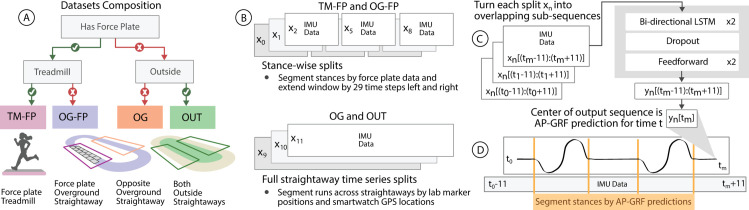
Data processing pipeline. **(A)** Collected data were partitioned into four datasets: TM-FP, OG-FP, OG, and OUT. **(B)** IMU data from TM-FP and OG-FP were divided into stance phases and extended by 29 frames before and after force plate contacts. IMU data from OG and OUT were divided into straightaways using motion capture or GPS coordinates. **(C)** Data windows, ${𝐱}_{n}$, were processed into smaller overlapping 22-frame sub-sequences and used as model inputs. For each output sub-sequence, ${𝐲}_{n}$, the model predicted AP-GRF at the mid-point, ${𝐭}_{m}$. **(D)** Estimated AP-GRF waveform is segmented into stance phases using a force threshold. TM = treadmill; OG = overground; FP = force plates; OUT = outdoors.

We partitioned collected data into four distinct settings: treadmill with force plates (TM-FP), overground running on force plates (OG-FP), overground running without force plates (OG), and outdoor overground running (OUT) (Fig 2A). In all settings, participants wore IMUs (Xsens DOT, Movella, Henderson, NV) on their pelvis and left lateral thigh, shank, and foot. Each IMU collected three dimensional local accelerations, angular velocities, and Euler angles at 120 Hz. IMU data were filtered with a bidirectional fourth-order Butterworth filter with a cutoff frequency (*f*_*c*_) of 50 Hz. We normalized all IMU data with the minimum-maximum value ranges from the TM-FP data used for generalized model training. Individual columns of three dimensional data, i.e., tri-axial acceleration and angular velocity, were normalized as groups, such that they retained relative magnitudes. We transformed the acceleration measurements of all IMUs into the same global reference frame. We extracted sagittal segment angles for the left thigh, shank, and foot based on previous work [25,30]. Specifically, we assumed the joints behaved as ideal hinges, meaning motion occurred primarily in the sagittal plane, and we aligned one IMU axis with the joint axis. To account for sensor drift, we reoriented the global IMU frame by aligning one axis with gravity and another with the joint axis, then calculated the sagittal segment angle as the rotation about the joint axis between the IMU’s local frame and the updated global frame.

For TM-FP and OG-FP data, three-dimensional GRFs were collected from an instrumented, split-belt treadmill and 13 floor-embedded force plates (Bertec Corp, Columbus, OH; 2 kHz). For speed calculation during in-lab overground running, a retro-reflective marker was placed on the sternal notch and recorded by an infrared motion capture system (Oqus and Miqus, Qualysis Corp, Gothenburg, Sweden; 200Hz). Participants wore additional markers on their left lower limb for data syncing purposes. Marker data were filtered with a bidirectional fourth-order Butterworth filter (*f*_*c*_ = 10 Hz), and GRFs were low-pass filtered (*f*_*c*_ = 50 Hz). To synchronize GRF and IMU data, a calibration maneuver was performed before each trial (three hard steps with the left foot followed by a forward and backward swing of the left leg). Markers worn on the left knee, shank, ankle, and foot were used to define foot-to-floor angle and were synchronized with GRFs (Visual 3D, C-Motion, Germantown, MD). Marker-derived foot-to-floor angle and IMU-derived sagittal foot angle were time-aligned by maximizing the cross-correlation between these angles during the calibration maneuver. The time delay identified from this foot angle cross-correlation was used to shift the GRF data to align with IMU data. Outdoors, participants wore a GPS-enabled smartwatch (Forerunner 255s, Garmin, Olathe, Kansas) to record location and speed. Smartwatch and IMU data were synced by aligning timestamps.

We used data with force plates (TM-FP and OG-FP) for training, fine-tuning, and testing our predictive models. Descriptive statistics of ground truth AP-GRF for a subset of training data is available in Table S1 Table in Supporting information. To facilitate control over data quantities during training and evaluation (e.g., number of strides), we first segmented TM-FP and OG-FP data into stance phases (Fig 2B) from initial contact to toe off using a 20 N vertical force threshold. We then extended the data window used for training to include 29 timesteps (0.242 s) before and after stance, which was the shortest observed swing phase in all of our data. Extending the data window to include swing phase data ensured predictive models could be applied to continuous time series data, including multiple stances when no segmentation by force plate is possible (e.g., for OG and OUT data).

OG and OUT data were segmented into straightaways to exclude data during turns. Straight sections of the lab track were identified using lab coordinates. The sternal marker was used to detect when the participant entered a straight section, and the marker’s horizontal position over time was used to calculate running speed. Straight sections of the outdoor track were identified using GPS coordinates, and the GPS measurements from the smartwatch were used to determine when the participant entered a straight section. Speed was also recorded from the smartwatch.

After stride or straightaway segmentation, each data window was further divided into sub-sequences of 22 frames (Fig 2C). These overlapping sub-sequences were used as model inputs. The time mid-point of the model output was selected as the estimated AP-GRF value. AP-GRF waveforms were created from the mid-points of all estimated sub-sequences.

By including data from swing phases into the training data, we enabled our neural network models to predict AP-GRF on continuous time series data comprising multiple strides. However, to also evaluate data quantities (i.e., stride counts) in OG and OUT straightaway data, and to not artificially improve AP-GRF prediction evaluation on TM-FP and OG-FP data by including trivial GRF predictions during swing phases, we segmented all predictions into stance phases using the AP-GRF predictions (Fig 2D). During the swing phases, AP-GRF predictions of our models were ∼ 0 %BW. We classified a series of predictions as a swing phase, if at least more than five consecutive predictions (∼40 ms) had a magnitude of ≤ 0.5 %BW and a slope of ≤ 0.25 %BW. We marked the beginning of a swing phase series as toe off (TO) and the end as initial contact (IC).

### Neural network models

To understand data requirements for maximizing accuracy of overground AP-GRF predictions, we compared different model training strategies using different datasets for training, fine-tuning, and testing (Table 1). The inputs to each model configuration were three-dimensional accelerations and angular velocities of the pelvis, and left thigh, shank, and foot. We also included the drift-corrected sagittal angles of the left thigh, shank, and foot. The output of each model configuration was the corresponding AP-GRF waveform (Fig 2D).

**Table 1:** Model training and fine-tuning configurations.

Model configuration	Training dataset	Fine-tuning dataset	Testing dataset	Inference dataset
GEN-TM	TM-FP (14)	-	OG-FP, split B (1)	OG (1) & OUT (1)
FTN-TM	TM-FP (14)	TM-FP (1)	OG-FP, split B (1)	-
FTN-OG	TM-FP (14)	OG-FP, split A (1)	OG-FP, split B (1)	OG (1) & OUT (1)
IND-OG	OG-FP, split A (1)	-	OG-FP, split B (1)	-

We define configurations as training, fine-tuning, testing data combinations and inference settings for our model. Numbers in parentheses indicate the number of participants taken from the dataset. Splits indicate if the data of one participant was further split into distinct training or testing datasets. GEN = generalized; IND = individual; TM = treadmill; OG = overground; FP = force plates; OUT = outdoors.

Models were evaluated using a leave-one-out cross validation approach. The generalized model, GEN-TM, was trained on treadmill data from 14 of 15 participants and tested on overground data from the excluded participant. The FTN-TM model fine-tuned GEN-TM using treadmill data from the test participant. The FTN-OG model fine-tuned GEN-TM using overground data from the test participant. IND-OG, an individual model, was trained only on the fine-tuning data of FTN-OG from the test participant.

The dataset sizes varied depending on the left-out test participant. Training and validation data for GEN-TM comprised all stances of 14 participants (7666 ± 191 stances from treadmill running across the left-out participants). They were split by a 9:1 ratio into training and validation sets. For fine-tuning, the training set of FTN-TM or FTN-OG included exactly eight stances from treadmill or overground running of the left-out participant, and an additional four stances as the validation set. The IND-OG training set was the same as the FTN-OG fine-tuning set, to compare performance after fine-tuning to a model trained only on individual data. The test set for all models comprised 54 ± 8 stances from overground running. We normalized data for each model using the minimum and maximum measurements from the respective training datasets. To preserve relative magnitudes in multi-dimensional signals, we normalized data in tri-axial groups. For example, each dimension of the three-dimensional acceleration signals in the training, validation, and test data was normalized using the global minimum and maximum values observed across all dimensions in the training set.

Our model architecture is a recurrent neural network with four hidden layers with the first two hidden layers being bidirectional long short term memory (LSTM) layers. We chose an LSTM network architecture because these have been shown to outperform feedforward neural networks for GRF predictions in related works [31,32]. We chose the number of layers through empirical search, then determined the following hyperparameters through a grid search approach. The LSTM had layers with 64 neurons each. We applied a dropout of 0.2 to the last LSTM layer. The final two hidden layers were fully connected layers with 128 and 64 neurons, respectively, followed by an output layer of size 1. We used the smooth L1 loss function, a learning rate of 0.001, and the AdamW optimizer. The GEN-TM model training ran for 40 epochs with a batch size of 32.

We also compared a range of fine-tuning datasets to characterize the relationship between the size of data from an individual versus prediction accuracy for that individual. Specifically, we evaluated using 2, 4, 8, 14, or 20 strides of treadmill or overground data. Fine-tuning involved updating all weights and biases of GEN-TM by continuing training for 15 epochs with a batch size of 16 using the fine-tune data sets. The IND-OG model was trained with a batch size of 1 to account for the small training set size. To evaluate model performance and estimation accuracy, we calculated the root mean squared error (RMSE) between the in-lab measures and the in-lab estimates.

### Prediction validation on overground data without force plates

In addition to comparing AP-GRF waveform estimation accuracy across models (on the TM-FP and OG-FP datasets), we estimated AP-GRF along the non-instrumented straightaway in the lab and during straight-line running out of the lab (OG and OUT datasets). We used the GEN-TM and FTN-OG model configurations to predict AP-GRF during OG and OUT conditions for five participants. We evaluated the plausibility of our predictions by calculating braking and propulsion impulses for each stance and comparing their correlation with running speeds to available ground-truth measurements from OG-FP.

Based on correlations shown in literature [33], we expect to observe that predicted braking and propulsion impulses demonstrate a linear correlation with speed. Hence, we fit linear models to braking and propulsion impulse estimates from GEN-TM predictions, FTN-OG predictions, and to available force plate measurements in OG-FP. We expected that FTN-OG predictions vary with speed more similarly to OG-FP than GEN-TM predictions for both OG and OUT.

Both the OG and OUT trials were collected at self-selected speeds, resulting in uneven data distributions with the most comfortable speeds of participants being overrepresented. To compensate for this data imbalance, for each participant, we binned the collected stances with 25 speed bins of size 0.1, ranging from 2 m/s to 4.5 m/s. Bins with less than three stances were discarded. We then fit a weighted linear model, such that, for a bucket i and all contained observations 𝐲_i_, the assigned weight was $w_{i}=\frac{1}{\sigma_{i}^{2}}$ [34]. We used RMSE to compare linear fits errors across the 2 m/s to 4.5 m/s bins. We compared the linear fits to braking and propulsion estimates of GEN-TM and FTN-OG to the linear fit to OG-FP force plate measurements. In this way, a lower RMSE indicated that braking and propulsion impulse relationships with speed matched more closely to observed trends when running across force plates in the lab. Across comparisons, we expected predictions of the fine-tuned model FTN-OG to match the observed speed-impulse relationship from ground truth OG-FP more closely than GEN-TM.

### Sensor Comparison

Finally, to understand the accuracy with a minimal sensor set suitable for regular outdoor use, we evaluated using only the IMU data of a single segment as input to the GEN-TM and FTN-OG model configurations. We compared RMSE of these single segment configurations to models with all the segments as input.

## Results

### Stance phase segmentation

As described in Materials and methods, we determined stance phase segments using AP-GRF estimates (Fig 2D). Compared to force plate-derived gait events for all time series data (TM-FP, OG-FP), the RMSE for estimate-derived gait events was 0.237 s for IC and 0.238 s for TO. These values match reported level-ground running IC and TO RMSE in related literature [14,35].

### Transfer from treadmill to overground

**Fig 3:**
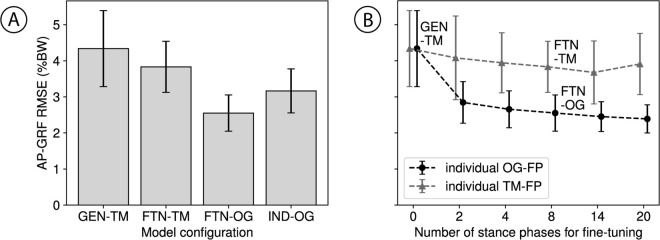
Prediction results. AP-GRF was estimated through leave-one-out cross validation across the 15 participants. Errors are presented as root mean squared error (RMSE). **(A)** RMSEs for a treadmill-trained generalized model (GEN-TM), fine-tuned GEN-TM with individual treadmill data (FTN-TM), fine-tuned GEN-TM with individual overground data (FTN-OG), and individual model with overground data (IND-OG). **(B)** Model performance when fine-tuned with an increasing number of stances from an individual’s treadmill data (TM-FP) or overground data (OG-FP). Observations that match model configurations in the bar plot in **(A)** are annotated.

GEN-TM achieved an average RMSE of 4.3 ± 1.1 %BW across the 15 participants (Fig 3). Furthermore, fine-tuning this treadmill-trained generalized model with individual treadmill data resulted in a more accurate model configuration (FTN-TM), with an RMSE of 3.8 ± 0.7 %BW. Fine-tuning GEN-TM with individual overground data led to even larger improvements, with the resultant model configuration (FTN-OG) achieving an error of 2.6 ± 0.5 %BW. Conversely, an individual model trained on the same data used to fine-tune FTN-OG (IND-OG) performed worse with an average error of 3.2 ± 0.6 %BW.

Fine-tuning GEN-TM with more individual-specific and setting-specific data improved overground braking and propulsion force predictions (Fig 3B). Fine-tuning GEN-TM with at least two strides of overground data improved FTN-OG to 2.8 ± 0.6 %BW. All numbers are provided in Table S2 Table in Supporting information.

### Transfer from lab to outdoors

**Fig 4:**
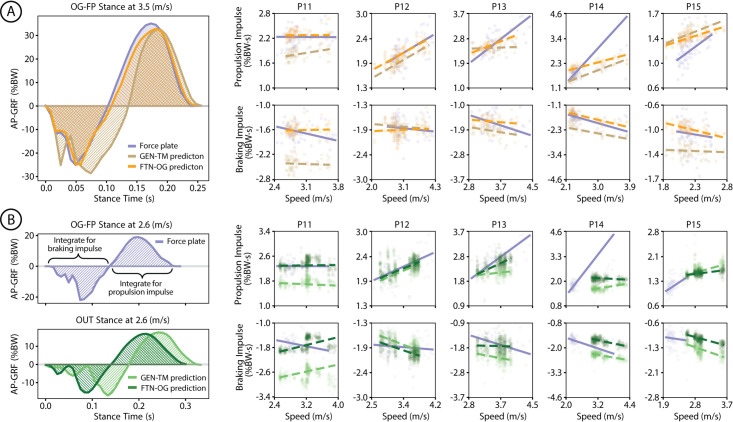
Outdoor comparison. Model inference on overground running without force plates. **(A)** Example AP-GRF waveforms from force plates during in-lab overground running (OG-FP; purple), and from the generalized model (GEN-TM; brown) and the overground fine-tuned model (FTN-OG; orange) predictions for lab-based running without force plates (OG). Braking and propulsion impulse are calculated from these waveforms. The linear relationship between speeds and braking/propulsion impulses are presented for five participants, with solid lines for ground truth force plate measurements and dashed lines for predictions. **(B)** A similar overview for outdoor running (OUT), depicting example predicted AP-GRF waveforms for GEN-TM (light green) and FTN-OG (dark green). The linear relationship between speeds and braking/propulsion impulses are again presented for five participants.

Braking and propulsion impulses during overground running derived from force plates (OG-FP) or derived from model predictions (GEN-TM, FTN-OG) varied with speed, in (Fig 4A) and out of the lab (Fig 4B). Linear models fit to approximate the relationship between lab-measured or estimation-derived impulse and speed showed lower RMSE between lab and FTN-OG compared to lab and GEN-TM for both OG and OUT predictions (Table 2). Specifically, for braking impulse, the RMSE between OG-FP and GEN-TM speed-braking linear model was 0.36 ± 0.25 %BW⋅s for OG and 0.47 ± 0.25 %BW⋅s for OUT. These errors decreased when comparing OG-FP and FTN-OG speed-braking linear models, with an RMSE of 0.16 ± 0.08 %BW⋅s for OG and 0.30 ± 0.13 %BW⋅s for OUT. The *R*^2^ values of the speed-braking linear models increased from GEN-TM to FTN-OG, in and out of the lab (OG: 0.09 ± 0.21 to 0.18 ± 0.26, OUT: 0.12 ± 0.30 to 0.27 ± 0.20). Similarly for propulsion impulse, the RMSEs between OG-FP and FTN-OG speed-propulsion linear models (OG: 0.44 ± 0.49 %BW⋅s, OUT: 0.53 ± 0.65 %BW⋅s) were lower than the RMSEs between OG-FP and GEN-TM speed-propulsion linear models (OG: 0.61 ± 0.50 %BW⋅s, OUT: 0.75 ± 0.69 %BW⋅s). The *R*^2^ values of the speed-propulsion linear models for OG increased from GEN-TM to FTN-OG (0.37 ± 0.30 to 0.43 ± 0.24), but not for OUT (0.04 ± 0.22 to 0.01 ± 0.16).

**Table 2:** Linear model comparison.

Impulse Metric	GEN-TM RMSE (%BW⋅s)	GEN-TM ${\text{R}}^{2}$	FTN-OG RMSE (%BW⋅s)	FTN-OG ${\text{R}}^{2}$
OG Braking	0.36 ± 0.25	0.09 ± 0.21	0.16 ± 0.08	0.18 ± 0.26
OG Propulsion	0.61 ± 0.50	0.37 ± 0.30	0.44 ± 0.49	0.43 ± 0.24
OUT Braking	0.47 ± 0.25	0.12 ± 0.30	0.30 ± 0.13	0.27 ± 0.20
OUT Propulsion	0.75 ± 0.69	0.04 ± 0.22	0.53 ± 0.65	0.01 ± 0.16

Linear models were created between running speed and AP-GRF impulse metrics, i.e. braking and propulsion, measured from force plates (OG-FP) or derived from model inference (GEN-TM, FTN-OG). Shown are the root mean square errors (RMSEs) between GEN-TM and FTN-OG linear model estimates and OG-FP linear model estimates, as well as the *R*^2^ values of the GEN-TM and FTN-OG linear fits. RMSEs were calculated using 25 points from each linear model uniformly sampled from 2 m/s to 4.5 m/s in 0.1 m/s increments. GEN = generalized; FTN = fine-tuned; TM = treadmill; OG = overground; FP = force plates; RMSE = root mean squared error; %BW = %bodyweight.

### Sensor comparison

**Fig 5:**
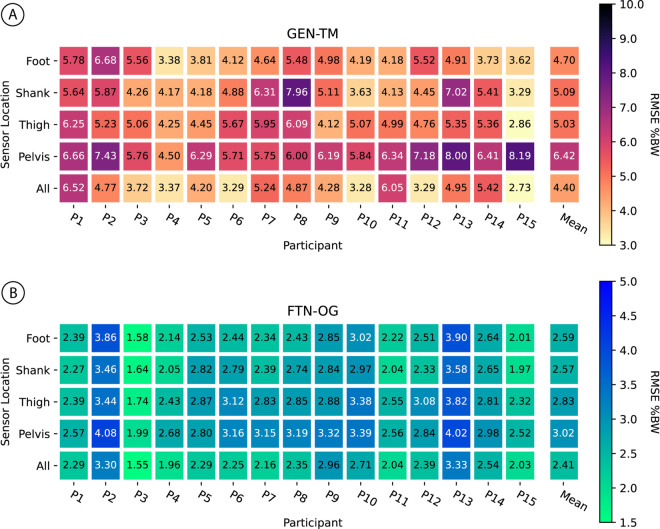
Single sensor comparison. Leave-one-out cross validation results when training and fine-tuning with IMU data from one segment, either foot, shank, thigh, or pelvis. The All row depicts errors when using all four IMUs. **(A)** GEN-TM prediction errors during overground running. **(B)** FTN-OG prediction errors during overground running. Values presented are root mean squared errors (RMSE). The last column is the mean error across 15 participants. GEN = generalized; FTN = fine-tuned; TM = treadmill; OG = overground.

We compared single sensor configurations for a minimal sensor set suitable for outdoor running. Using the IMU data of any single segment as the sole input to GEN-TM (Fig 5A) or FTN-OG (Fig 5B) resulted in a higher RMSE compared to using the four IMUs as model inputs to predict overground AP-GRF. Averaged across participants, GEN-TM RMSE was 4.40 ± 1.09 %BW for all IMUs, 4.70 ± 0.92 %BW for foot only, 5.09 ± 1.25 %BW for shank only, 5.03 ± 0.84 %BW for thigh only, and 6.42 ± 0.93 %BW for pelvis only. For FTN-OG, RMSE was 2.41 ± 0.48 %BW for all IMUs, 2.59 ± 0.60 %BW for foot only, 2.57 ± 0.53 %BW for shank only, 2.83 ± 0.50 %BW for thigh only, and 3.02 ± 0.54 %BW for pelvis only.

## Discussion

The primary goals of this work were to assess transferability of a generalized model trained on IMU data from 14 participants during treadmill running to predict individual overground AP-GRF of a left-out participant, then to characterize how fine-tuning with individual data improves predictions, and finally, to evaluate fine-tuned models for outdoor running.

Evaluation of GEN-TM predictions on lab-based overground data revealed an RMSE of 4.3 %BW. This level of accuracy is consistent with previous studies using machine learning to predict AP-GRF with IMU data [17], with models trained and tested within a single running environment, such as on the treadmill (7.0 %BW [20]) or overground (4.3 %BW [26]). The performance of GEN-TM on overground data, collected at self-selected speeds and cadences, suggests that a generalized model trained on treadmill data can transfer to unseen runners and unseen overground running conditions, achieving accuracy within the expectations set by prior work. Future work could extend estimation models to running on varied surfaces, such as trails and inclines.

As expected, fine-tuning GEN-TM with an individual’s overground data yielded the most accurate predictions of overground AP-GRF. Using eight strides of individual overground data for fine-tuning in the FTN-OG model configuration reduced the RMSE from 4.3 to 2.6 %BW. Notably, even as few as two overground strides reduced error to 2.8 %BW. This finding suggests that critical information about an individual’s kinetics may be effectively captured with minimal data. While amassing an individual training dataset is time- and resource-intensive, fine-tuning a pre-trained generalized model with a few overground strides from a new participant balances collection requirements (less than 10 passes over force plates if collecting one stride per pass) with estimation accuracy improvement. This approach enhances the feasibility of creating individualized models for braking and propulsion estimation during overground running.

Even when overground force plates are unavailable, this study demonstrated the potential of fine-tuning with individual treadmill data to improve overground AP-GRF predictions. Specifically, fine-tuning GEN-TM with eight strides of individual treadmill data in the FTN-TM model configuration reduced the RMSE from 4.3 to 3.8 %BW. This result suggests that individual movement patterns are important to model accuracy, regardless of whether the data originates from treadmill or overground running. Although all running involves a braking phase and propulsion phase, the exact spatiotemporal, kinematic, kinetic, and musculotendon patterns executed with each step differ between runners [36]. Fine-tuning with individual-specific data allows the model to better learn and adapt to the unique biomechanical relationships of each runner, enhancing braking and propulsion estimation accuracy over a generalized model.

Comparing the generalized and fine-tuned model configurations to a model trained exclusively on individual overground data highlighted the importance of large training datasets as well as the inclusion of individual- and setting-specific data in model training. IND-OG, trained on the same overground data which was used to fine-tune GEN-TM in the FTN-OG model configuration, performed worse than FTN-OG (RMSE 3.2 vs. 2.6 %BW) but better than FTN-TM (3.8 %BW) and GEN-TM (4.3 %BW). Including data from a variety of runners at a variety of speeds and cadences in the generalized model before fine-tuning with minimal overground data enabled better predictions compared to training an individual model with minimal overground data alone. This finding reinforces the efficiency of fine-tuning for predicting overground AP-GRF and underscores the value of leveraging pre-existing datasets [37,38] to train generalized models that can then be individually fine-tuned.

For validation on outdoor and lab-based overground data without force plates, we leveraged GEN-TM and FTN-OG model configurations and compared observed trends to ground truth force plate observations. Consistent with in-lab comparisons, FTN-OG provided estimates that were better aligned with lab data than GEN-TM, again highlighting the benefit of fine-tuning with individual data. Specifically, FTN-OG predictions outdoors followed the expected linear trends between braking/propulsion impulses and speed more closely than GEN-TM. While the *R*^2^ values of these speed-impulse linear models mostly increased with fine-tuning, they ranged from 0.01 to 0.43, suggesting that, in some cases, not much of the variation in braking or propulsion impulse was explained by changes in speed. This limitation highlights the need for additional strategies to validate model performance, especially in real-world settings. Although direct AP-GRF measurements remain the gold standard for model validation, they are challenging to obtain with current technology. There is growing interest in developing methods to relate and compare outdoor observations with in-lab data [4]. Large indoor overground tracks with instrumented straightaways [13] will be valuable for future validation.

Minimizing the number of sensors required for accurate estimations is likely essential to real-world adoption. To address this, we evaluated single-sensor configurations to identify a minimal sensor set suitable for outdoor running. Among single-segment configurations, FTN-OG demonstrated average errors below 3 %BW when using an IMU on the thigh, shank, or foot alone. However, using all four IMUs (pelvis, left thigh, shank, and foot) as model inputs resulted in better accuracy than using any single sensor. This trend was true for both GEN-TM and FTN-OG model configurations. The accuracy differences between participants suggests that some individuals may require more sensors for optimal braking and propulsion predictions. While this study did not collect IMU data from the wrist, given the widespread use of smartwatches by runners, exploring methods that leverage smartwatch IMU data in future research could enable more accessible data collection, load estimation, and injury prediction [39]. For example, recent work has shown that mapping insole data to a wrist-worn activity tracker during a calibration enables tibial load estimation using only a wrist-worn sensor [40]. As a next step, this research could consider a similar approach and investigate mapping lower-limb IMU data to wrist-worn IMU data, potentially enabling more ubiquitous and practical biomechanical analysis outside the lab.

There were several limitations in this study. The datasets included only level-ground, straight-line running, and training and fine-tuning data requirements during more variable running conditions are unknown. Although the self-selected speeds and cadences during overground running introduced some step-to-step variability, all overground running was conducted on constrained oval tracks, limiting variability in movement patterns. Further, the small size and curvature of the indoor lab track may have limited running speeds and limited the generalization of the model to faster speeds. Differences in ground stiffness between the running environments may have influenced AP-GRF magnitudes [41], as well as air resistance during outdoor running, which was not taken into account. Participants wore their typical running shoes to ensure comfort throughout the study, but differences in footwear, including shoe stiffness and stack height, may have influenced AP-GRF magnitudes.While this study used similar participant numbers as other estimation work [2,3], expanding the dataset to include more runners will be more representative and robust and enable subgroup analysis based on foot strike pattern, gender, etc. We collected IMU data for a relatively short duration (less than one hour of running), and model performance over longer timescales is unknown. Additionally, we collected only unilateral IMU data from the left leg for all participants, without accounting for limb dominance. Incorporating bilateral measurements may improve model performance and enable analysis of limb asymmetries. Lastly, IMU hardware used in this study limited data collection to a sampling frequency of 120 Hz. While prior work has estimated running metrics at 100 Hz [14,31], studies suggest that sampling frequencies above 200 Hz may be necessary for more reliable orientation measurements[42] and kinematic estimation [43], and frequencies upwards of 500 Hz may be required to best capture kinetic metrics [43].

Several directions could further improve wearable-based gait analysis and its practical impact. Developing robust sensor calibration strategies will be important [44] to eliminate the dependency on sensor-to-segment alignment [45,46], as sensors may shift due to sweat, soft tissue motion, or donning variation across days, and signals may drift over time [47]. Future work could also explore providing runners with feedback on their braking and propulsion forces during outdoor running, similarly to spatiotemporal metrics [48,49], with applications in performance and injury prevention. Coordinated propulsion and braking have been linked to greater running economy and adaptability [50], and the nature of this coordination may reflect individual gait strategies. For example, one study found that runners with a higher duty factor and lower cadence used a propulsion-driven strategy, while those with a lower duty factor and higher cadence exhibited gait mechanics more consistent with the spring-mass system [51]. Monitoring AP-GRF may therefore offer valuable insight for performance optimization and gait retraining. From an injury perspective, an exploratory study found that real-time biofeedback during treadmill running reduced peak braking forces among recreational runners [52]. Translating such feedback-based gait retraining to real-world settings using IMU-based braking force estimates could broaden its accessibility. Recent advancements have shown the feasibility of continuously estimating GRFs using IMU data and computationally efficient models [18,53]. Future studies could integrate these real-time kinetic estimations as real-time feedback to the runner and monitor their gait changes over time. Equipping running coaches with data-driven tools to complement their existing feedback frameworks [54,55] is key to maximizing runner performance while minimizing the risk of running-related injuries.

## Conclusion

Leveraging a dataset from treadmill and overground running conditions, we observed that a generalized IMU data-driven model trained on treadmill running can effectively estimate braking and propulsion forces during overground running. Fine-tuning with eight strides of individual-specific data improved predictions. Accordingly, when extended to outdoor overground running, the fine-tuned model predicted forces across speeds that better aligned with lab measurements than the generalized model’s predictions. These findings highlight the value of combining generalized and individualized approaches to improve the transferability and accuracy of kinetic estimation. While this work focused on straight level-ground running, future work should investigate more diverse running conditions to further contribute toward translating in-lab kinetic estimates from IMU data to real-world settings.

## Supporting information

S1 Table Descriptive statistics of AP-GRF across varying speed and cadence conditions (N = 5). This table presents peak braking and propulsion force magnitudes across speed and cadence combinations from a subset of five participants (P11-P15), measured from force plates during treadmill running. Magnitudes are reported in %bodyweight (%BW). Cadence was manipulated using a metronome. To ensure steady speed and metronome match, the middle 30 strides per condition per participant were used to generate these values.
(PDF)

S2 Table Fine-tuning iterations. This table provides the numbers for our results in Fig 3B in the manuscript. Summarized are the prediction error improvements as fine-tuning dataset sizes increased. FTN = fine-tuned; TM = treadmill; OG = overground; FP = force plates; RMSE = root mean squared error; %BW = %bodyweight.
(PDF)
