# Supplementary material for: Estimating braking and propulsion forces during overground running in and out of the lab
Source: PLoS One. 2025 Sep 4;20(9):e0330042. doi: 10.1371/journal.pone.0330042 (PMC12410772; doi:10.1371/journal.pone.0330042)
Supplement: S1 Table — This table presents peak braking and propulsion force magnitudes across speed and cadence combinations from a subset of five participants (P11-P15), measured from force plates during treadmill running. Magnitudes are reported in %bodyweight (%BW). Cadence was manipulated using a metronome. To ensure steady speed and metronome match, the middle 30 strides per condition per participant were used to generate these values. (PDF) [file pone.0330042.s001.pdf]

Estimating braking and propulsion forces during overground running in and out of the lab

Lauren M. Baker[1], Fabian C. Weigend[1¶], Krithika Swaminathan[1¶], Daekyum Kim[1,2], Andrew Chin[1], Daniel E. Lieberman[3], Conor J. Walsh[1*]

1 John A. Paulson School of Engineering and Applied Sciences, Harvard University, Boston, MA, United States of America
2 School of Mechanical Engineering, Korea University, Seoul, Republic of Korea
3 Department of Human Evolutionary Biology, Harvard University, Cambridge, MA, United States of America

¶These authors contributed equally to this work.
* walsh@seas.harvard.edu

Supporting information

Table S1. Descriptive statistics of AP-GRF across varying speed and cadence conditions (N=5).

Speed (m/s)	Cadence (steps/min)	Peak braking (%BW)		Peak propulsion (%BW)	
		Mean ± std	[Min, Max]	Mean ± std	[Min, Max]
2	170	-21.4 ± 5.3	[−35.4, −12.4]	16.2 ± 3.7	[11.3, 28.2]
2.5	170	-27.2 ± 7.6	[−51.1, −12.5]	20.9 ± 3.5	[15.0, 31.7]
3	170	-30.7 ± 6.4	[−49.0, −18.4]	25.9 ± 4.2	[18.1, 37.1]
3.5	170	-36.5 ± 8.8	[−65.6, −23.1]	29.8 ± 4.1	[24.0, 40.4]
4	170	-43.5 ± 15.1	[−94.9, −24.2]	33.9 ± 4.7	[26.0, 46.1]
3	150	-30.7 ± 5.6	[−43.4, −18.8]	26.5 ± 2.2	[21.0, 33.9]
3	160	-31.4 ± 6.0	[−52.6, −20.4]	26.7 ± 3.5	[20.7, 36.5]
3	180	-31.1 ± 9.1	[−66.2, −19.1]	25.0 ± 4.0	[19.7, 37.3]
3	190	-30.0 ± 9.0	[−66.2, −17.2]	24.2 ± 4.2	[17.2, 35.3]

This table presents peak braking and propulsion force magnitudes across speed and cadence combinations from a subset of five participants (P11-P15), measured from force plates during treadmill running. Magnitudes are reported in %bodyweight (%BW). Cadence was manipulated using a metronome. To ensure steady speed and metronome match, the middle 30 strides per condition per participant were used to generate these values.
