# Supplementary material for: Estimating braking and propulsion forces during overground running in and out of the lab
Source: PLoS One. 2025 Sep 4;20(9):e0330042. doi: 10.1371/journal.pone.0330042 (PMC12410772; doi:10.1371/journal.pone.0330042)
Supplement: S2 Table — This table provides the numbers for our results in Fig 3B in the manuscript. Summarized are the prediction error improvements as fine-tuning dataset sizes increased. FTN = fine-tuned; TM = treadmill; OG = overground; FP = force plates; RMSE = root mean squared error; %BW = %bodyweight. (PDF) [file pone.0330042.s002.pdf]

Estimating braking and propulsion forces during overground running in and out of the lab

Lauren M. Baker[1], Fabian C. Weigend[1¶], Krithika Swaminathan[1¶], Daekyum Kim[1,2], Andrew Chin[1], Daniel E. Lieberman[3], Conor J. Walsh[1*]

1 John A. Paulson School of Engineering and Applied Sciences, Harvard University, Boston, MA, United States of America
2 School of Mechanical Engineering, Korea University, Seoul, Republic of Korea
3 Department of Human Evolutionary Biology, Harvard University, Cambridge, MA, United States of America

¶These authors contributed equally to this work.
* walsh@seas.harvard.edu

Supporting information

Table S2. Fine-tuning iterations.

Number of fine-tuning stance phases	FTN-TM and OG-FP RMSE (%BW)	FTN-OG and OG-FP RMSE (%BW)
0	4.3 ± 1.1	4.3 ± 1.1
2	4.1 ± 1.2	2.8 ± 0.6
4	3.9 ± 0.8	2.7 ± 0.5
8	3.8 ± 0.7	2.6 ± 0.5
14	3.7 ± 0.9	2.5 ± 0.4
20	3.9 ± 0.8	2.4 ± 0.4

This table provides the numbers for our results Fig 3B in the manuscript. Summarized are the prediction error improvements as fine-tuning dataset sizes increased. FTN = fine-tuned; TM = treadmill; OG = overground; FP = force plates; RMSE = root mean squared error; %BW = %bodyweight.
